# The King's Fund and Lord Rosebery

**Published:** 1912-04-13

**Authors:** Savile Crossley, John G. Griffiths

**Affiliations:** Honorary Secretary.; Honorary Secretary.


*To the Editor of* The Hospital.

### THE KING'S FUND AND LORD ROSEBERY.

*To the Editor of* The Hospital.

Sir,—Lord Rosebery, as President of the Royal National Hospital for Consumption, Ventnor, having for the second time publicly complained of the action of the Council of King Edward's Hospital Fund in not making a grant towards that institution, it may be thought desirable that the Fund should give the reasons for its action—reasons which have hitherto only been indicated indirectly in the annual report of the Convalescent Homes Committee of the Fund.

These reasons are very simple. They do not imply any doubts as to the efficiency of the hospital, but are based only upon its financial position. The primary object of the Fund is to assist hospitals which are in need. The accounts furnished by the Ventnor Hospital in support of its three recent applications to the Fund showed excesses of income over expenditure of £3,331 in 1907, £7,152 in 1909, and £958 in 1910. It is true that these surpluses were due to legacies, and that in the case of a hospital which receives exceptional legacies in a given year, or which has so little free capital that the investment of any unspent legacies is a matter of financial urgency, the existence of such a surplus would not be decisive against a grant being made. But in the case of this hospital, the average surplus for the six years (1905 to 1910) for which the figures have been submitted to the Fund was £2,119 on an average annual expenditure of £13,356, while in the year last mentioned the free capital amounted to £63,428, besides endowments of £8,945. In only one of these years did the figures show a deficit, and in the year in which those accounts were published the hospital did not apply to the Fund. Any grant from the Fund would therefore have been equivalent to a grant to capital, and under these circumstances the King's Fund considered that the moneys entrusted to it for distribution could be better employed in assisting institutions which were in greater need, or in increasing (as it has been able to do elsewhere) the amount of sanatorium accommodation available for London patients.

All this Lord Rosebery could have ascertained for himself if his other numerous engagements had permitted him to attend any of the meetings of the King's Fund, of which he has been a member since 1908.—We are, Sir, your obedient servants,

(Signed) Savile Crossley, John G. Griffiths, } Honorary Secretaries.

